# Effect of 6-month vs. 8-month regimen on retreatment success for pulmonary TB

**DOI:** 10.5588/ijtld.22.0357

**Published:** 2022-12-01

**Authors:** J. Izudi, L. A. Sheira, F. Bajunirwe, S. I. McCoy, A. Cattamanchi

**Affiliations:** 1 Department of Community Health, Mbarara University of Science and Technology, Mbarara, Uganda; 2 Infectious Diseases Institute, College of Health Sciences, Makerere University, Kampala, Uganda; 3 African Population and Health Research Center, Nairobi, Kenya; 4 Division of Epidemiology, School of Public Health, University of California, Berkeley, CA, USA; 5 Division of Pulmonary and Critical Care Medicine and Center for Tuberculosis, University of California San Francisco, CA, USA

Dear Editor,

The optimal regimen for the retreatment of pulmonary TB (PTB) is unclear. Until 2017, the WHO recommended an 8-month regimen consisting of rifampicin (R, RIF), isoniazid (H, INH), pyrazinamide (Z), ethambutol (E) and streptomycin (S) (or 2RHZES/1RHZE/5RHE).^1^ The WHO recently issued a conditional recommendation to replace the 8-month regimen with the standard 6-month regimen (2RHZ/4RH) for patients without RIF resistance (and preferably also INH resistance) identified using molecular testing. This conditional recommendation was based on systematic reviews that identified a low pooled treatment success rate (TSR) of 68% for retreatment of TB.^2^,^3^ However, head-to-head comparisons of the two regimens are needed to strengthen the recommendation and enhance uptake.

We therefore designed the Anti-TB Regimen (ATTIRE) study, a quasi-experimental study to evaluate the effect of the 6- vs. 8-month treatment regimen on TSR and sputum smear conversion (SSC) for retreatment of bacteriologically confirmed PTB in people aged ≥15 years, in Kampala, Uganda. The ATTIRE study abstracted data from six TB clinics across Kampala Capital City Authority (KCCA) health facilities between November 2021 and February 2022. The dataset is deposited elsewhere.^4^ The clinics provide TB diagnostic and treatment services following national guidelines. Baseline, follow-up and treatment outcome data are recorded in a TB unit register. People undergoing retreatment received sputum smear monitoring at 3, 5 and 8 months under the 8-month regimen, or at 2, 5 and 6 months under the 6-month regimen. Sputum smear monitoring establishes treatment response through conversion from positive to negative. We included people with TB aged ≥15 years treated between January 2012 and December 2021. We excluded people with confirmed or intermediate RIF resistance (as they should receive a second-line TB treatment regimen) and people with unknown RIF resistance status. The exposed group included people who received the 6-month treatment regimen, whereas the unexposed group included people who received the 8-month regimen. The primary outcome was TSR (yes or no) measured by cure or treatment completion.^5^ The secondary outcome was SSC at the follow-up time points. Baseline covariates included age, sex, level of health facility, type of TB disease, HIV status, body weight (kg), *Mycobacterium tuberculosis* (MTB) load, RIF resistance status, treatment delivery model and treatment supporter availability. We summarised categorical data using frequencies and percentages and numerical data using means with standard deviations. We applied doubly-robust estimation, a statistical approach for causal analysis that combined exposure and outcome regression models^6^ to ensure unbiased cause-effect measure, provided one of the models was correctly specified, but not both.^7^ First, we fitted separate outcome regression models for the 6- and 8-month treatment regimen groups containing all the covariates. We used the two models to estimate the exposure effect, given the covariate values for all the participants. The predicted values for the exposed group were therefore the counterfactual outcomes had all participants received the 6-month treatment regimen, and the predicted values for the unexposed group were the counterfactual outcomes had all participants received the 8-month treatment regimen. Second, we fitted an exposure regression model to estimate propensity scores. We used the inverse of propensity scores to weigh participants in the 6-month treatment regimen group, and the inverse of one minus the propensity score to weigh those in the 8-month treatment regimen group.^8^ Weighting created two pseudo-populations, one for each group where all covariates are balanced; this was confirmed using a standardised mean difference of <0.10.^9^ Finally, we combined estimates from the two models and reported the average causal effect using marginal means of potential outcomes as marginal odds ratio (mOR) and 95% confidence intervals (CIs).^8^ We received ethical approval from Clarke International University Research Ethics Committee, Kampala, Uganda (CLARKE-2021-101), and administrative clearance from the KCCA Directorate of Public Health and Environment, Kampala, Uganda (DPHE/KCCA/1301).

We analysed data for 427 participants, with similar age distribution across both treatment regimens (Table 1). TSR was 49.6% overall, and higher in the 6-month regimen group than in the 8-month regimen group (52.2% vs. 46.7%). More participants in the 6-month regimen group achieved SSC than in the 8-month treatment regimen group. Causal effect analysis showed that the 6-month treatment regimen significantly improved TSR compared to the 8-month treatment regimen (mOR 1.27, 95% CI 1.06–1.52). However, SSC rates were similar at 2/3 months (mOR 1.01, 95% CI 0.91–1.12), 5 months (mOR 0.96, 95% CI 0.88–1.04) and 6/8 months (mOR 0.83, 95% CI 0.19–3.51). A TSR of 52.2% for the 6-month regimen was lower than the national TSR of 65% for people undergoing retreatment for PTB (excluding relapse cases), and slightly lower than the 68.1% TSR for retreatment of PTB in eastern Uganda.^10^ However, the TSR remains distant from the WHO-desired target of ≥90%. Accordingly, measures should be taken by both the district and national TB control programmes to improve TSR among people with retreatment PTB as it fuels drug resistance.

**Table 1:** Sociodemographic characteristics of study participants.

Variables	Level	Overall (*n* = 427) *n* (%)	8-month treatment regimen (*n* = 197) *n* (%)	6-month treatment regimen (*n* = 230) *n* (%)	*P* value
Level of health facility	Health Centre III	200 (46.8)	83 (42.1)	117 (50.9)	0.088
	Health Centre IV	227 (53.2)	114 (57.9)	113 (49.1)	
Study sites	A	79 (18.5)	26 (13.2)	53 (23.0)	0.01
	B	50 (11.7)	34 (17.3)	16 (7.0)	
	C	112 (26.2)	51 (25.9)	61 (26.5)	
	D	46 (10.8)	21 (10.7)	25 (10.9)	
	E	125 (29.3)	58 (29.4)	67 (29.1)	
	F	15 (3.5)	7 (3.6)	8 (3.5)	
Sex	Female	123 (28.8)	51 (25.9)	72 (31.3)	0.261
	Male	304 (71.2)	146 (74.1)	158 (68.7)	
Age groups, years	15–24	65 (15.2)	28 (14.2)	37 (16.1)	0.225
	25–34	162 (37.9)	85 (43.1)	77 (33.5)	
	35–44	121 (28.3)	48 (24.4)	73 (31.7)	
	45–54	60 (14.1)	29 (14.7)	31 (13.5)	
	≥55	19 (4.4)	7 (3.6)	12 (5.2)	
	Mean ± SD	34.6 ± 10.8	34.4 ± 11.0	34.8 ± 10.6	0.695
Risk categories	Diabetic patient	1 (0.2)	0 (0.0)	1 (0.4)	0.200
	Health worker	1 (0.2)	0 (0.0)	1 (0.4)	
	Mentally ill	1 (0.2)	0 (0.0)	1 (0.4)	
	Miner	12 (2.8)	6 (3.0)	6 (2.6)	
	TB contact	294 (68.9)	126 (64.0)	168 (73.0)	
	Tobacco user	83 (19.4)	48 (24.4)	35 (15.2)	
	Uniformed personnel	35 (8.2)	17 (8.6)	18 (7.8)	

The improved TSR of the 6-month regimen compared to the 8-month regimen group might be attributed to biological or socially plausible factors. Biologically, exposure to streptomycin, which is associated with higher rates of toxicity, perhaps compromised treatment adherence in the latter group. Socially, the latter regimen has a slightly longer treatment duration, which may contribute to lower treatment adherence due to drug fatigue.^11^ The finding of a similar effect on SSC suggests both regimens are effective. However, the improvement in SSC in the first 2 months is probably explained by the low toxicity rate associated with the 6-month compared to the 8-month regimen; hence, treatment adherence was better.^12^ The reduced SSC thereafter might be explained by the few patients who did not achieve SSC.

Overall, we found the 6-month regimen achieved a better TSR than the 8-month regimen (Table 2). Therefore, future treatment should continue with the 6-month regimen. Further research is needed to determine the prevalence of INH resistance and cost-effectiveness of routine testing for INH resistance. This should be done at different prevalence thresholds, as patients with INH monoresistance should be treated with levofloxacin, RIF, ethambutol and pyrazinamide for 6 months. Future research should also assess whether – in the absence of RIF resistance – treatment can be further shortened to 4 months.^13^

**Table 2:** Clinical characteristics of study participants.

Variables	Level	Overall (*n* = 427) *n* (%)	8-month treatment regimen (*n* = 197) *n* (%)	6-month treatment regimen (*n* = 230) *n* (%)	*P* value
MTB bacilli load	+ (10–99 AFB/100 fields)	174 (40.7)	81 (41.1)	93 (40.4)	0.418
	++ (1–10 AFB/field)	197 (46.1)	86 (43.7)	111 (48.3)	
	+++ (>10 AFB/field)	56 (13.1)	30 (15.2)	26 (11.3)	
MTB/RR-TB status using Xpert testing	MTB detected/RR-TB not detected	191 (44.7)	87 (44.2)	104 (45.2)	0.904
	MTB not detected/RR-TB not detected	236 (55.3)	110 (55.8)	126 (54.8)	
Baseline weight, kg	Mean ± SD	51.3 ± 16.6	51.6 ± 16.8	51.0 ± 16.4	0.701
HIV status	Negative	158 (37.0)	66 (33.5)	92 (40.0)	<0.001
	Positive	132 (30.9)	45 (22.8)	87 (37.8)	
	Unknown	137 (32.1)	86 (43.7)	51 (22.2)	
Treatment model	Digital community DOT	47 (11.0)	18 (9.1)	29 (12.6)	0.459
	Health facility-based	363 (85.0)	172 (87.3)	191 (83.0)	
	Non-digital community DOT	17 (4.0)	7 (3.6)	10 (4.3)	
Sputum smear test at Month 2/3	No	48 (11.2)	30 (15.2)	18 (7.8)	0.024
	Yes	379 (88.8)	167 (84.8)	212 (92.2)	
Sputum smear test at Month 5	No	55 (12.9)	33 (16.8)	22 (9.6)	0.039
	Yes	372 (87.1)	164 (83.2)	208 (90.4)	
Sputum smear test at Month 6/8	No	76 (17.8)	42 (21.3)	34 (14.8)	0.102
	Yes	351 (82.2)	155 (78.7)	196 (85.2)	
Treatment supporter available	No	81 (19.0)	41 (20.8)	40 (17.4)	0.438
	Yes	346 (81.0)	156 (79.2)	190 (82.6)	
Treatment success	No	215 (50.4)	105 (53.3)	110 (47.8)	0.303
	Yes	212 (49.6)	92 (46.7)	120 (52.2)	
Sputum outcome at Month 2/3 (*n* = 379)*	Positive	169 (44.6)	77 (46.1)	92 (43.4)	0.598
	Negative	210 (55.4)	90 (53.9)	120 (56.6)	
Sputum outcome at Month 5 (*n* = 372)*	Positive	163 (43.8)	73 (44.5)	90 (43.3)	0.810
	Negative	209 (56.2)	91 (55.5)	118 (56.7)	
Sputum outcome at Month 6/8 (*n* = 351)*	Positive	8 (2.3)	3 (1.9)	5 (2.6)	0.701
	Negative	343 (97.7)	152 (98.1)	191 (97.4)	
Treatment outcomes	Cured	203 (47.5)	86 (43.7)	117 (50.9)	0.035
	Treatment completed	9 (2.1)	6 (3.0)	3 (1.3)	
	Treatment failed	144 (33.7)	62 (31.5)	82 (35.7)	
	Dead	14 (3.3)	6 (3.0)	8 (3.5)	
	Lost to follow-up	24 (5.6)	17 (8.6)	7 (3.0)	
	Transfer-out	33 (7.7)	20 (10.2)	13 (5.7)	

* The data analysis was restricted to participants who received sputum smear examination at respective time points and to participants with pulmonary bacteriologically confirmed TB.

SD = standard deviation; MTB = *Mycobacterium tuberculosis*; AFB = acid-fast bacilli; RR-TB = rifampicin-resistant TB; DOT = directly observed therapy.
